# Evaluation of the Bladder Stimulation Technique to Collect Midstream Urine in Infants in a Pediatric Emergency Department

**DOI:** 10.1371/journal.pone.0152598

**Published:** 2016-03-31

**Authors:** Antoine Tran, Clara Fortier, Lisa Giovannini-Chami, Diane Demonchy, Hervé Caci, Jonathan Desmontils, Isabelle Montaudie-Dumas, Ronny Bensaïd, Hervé Haas, Etienne Berard

**Affiliations:** 1 Hôpitaux Pédiatriques de Nice CHU-Lenval, Nice, France; 2 Service de Néphrologie Pédiatrique, CHU de Nice, Archet 2, Nice, France; Université Paris Descartes; AP-HP, Groupe Hospitalier Cochin-Saint-Vincent-de-Paul, FRANCE

## Abstract

**Objective:**

Midstream clean-catch urine is an accepted method to diagnose urinary tract infection but is impracticable in infants before potty training. We tested the bladder stimulation technique to obtain a clean-catch urine sample in infants.

**Materials and methods:**

We included 142 infants under walking age who required a urine sample in a cross- sectional study carried out during a 3-months period, from September to November 2014, in the emergency department of the University Children’s Hospital of Nice (France). A technique based on bladder stimulation and lumbar stimulation maneuvers, with at least two attempts, was tested by four trained physicians. The success rate and time to obtain urine sample within 3 minutes were evaluated. Discomfort (EVENDOL score ≥4/15) was measured. We estimated the risk factors in the failure of the technique. Chi-square test or Fisher’s exact test were used to compare frequencies. T-test and Wilcoxon test were used to compare quantitative data according to the normality of the distribution. Risk factors for failure of the technique were evaluated using a multivariate logistic regression model.

**Results:**

We obtained midstream clean-catch urine in 55.6% of infants with a median time of 52.0 s (10.0; 110.0). The success rate decreased with age from 88.9% (newborn) to 28.6% (>1 y) (p = 0.0001) and with weight, from 85.7% (<4kg) to 28.6% (>10kg) (p = 0.0004). The success rate was 60.8% for infants without discomfort (p<0.0001). Heavy weight and discomfort were associated with failure, with adjusted ORs of 1.47 [1.04–2.31] and 6.65 [2.85–15.54], respectively.

**Conclusion:**

Bladder stimulation seems to be efficient in obtaining midstream urine with a moderate success rate in our study sample. This could be an alternative technique for infants before potty training but further randomized multicenter studies are needed to validate this procedure.

## Introduction

Urinary tract infection (UTI) is common in children [1–4]. Overall, 3–5% of young, febrile children have a UTI, including 5–7% of those “without a source of fever”. The risk for UTI before the age of 2 years is approximately 1–4% in boys and 3–8% in girls [5–7]. The risk for a subsequent UTI varies between 12–30% in the 6–12 months following the initial episode [8–11]. A quick diagnosis and appropriate treatment may avoid morbidity and long-term sequelae (e.g., hypertension, renal scarring, poor renal growth, recurrent pyelonephritis, impaired glomerular function) [12–16].

According to the American Academy of Pediatrics (AAP) [17,18], midstream clean-catch urine (CCU) is an accepted method to diagnose UTI. However, this method is impractical in infants before potty training. Alternatively, urine samples are collected using sterile bags. This is an easier technique, albeit time consuming, with 63% specificity [19,20], with the strong inconvenience of a high rate of contamination: from 40% to 62.8% according to published studies [21,22]. Other valuable but invasive techniques are suprapubic aspiration (SPA) and bladder catheterization. These reduce the risk of urethral or skin contamination [23] but are invasive and painful procedures [24]. As a result, the 2011 AAP guidelines recommend that perineal bags should be used in children who do not appear to be unwell and have a low likelihood of UTI, as a screening step to decide whether to perform bladder catheterization or SPA (recommended to obtain a sample for urine culture in children with a positive urinalysis from a perineal bag).

The natural voiding pattern in newborns [25] is characterized by small and frequent voids. Preterm and full term newborns void once an hour (20–24 times per day) and the number of voids decreases from this age to 4–7 voids per day after toilet training. Furthermore, in the case of a sudden increase in abdominal pressure (such as abdominal bumping), the pudendal nerve efferents are responsible for the guarding reflex to avoid micturition. This reflex is progressively controlled after 2 years of age but is not effective in newborns. Recently, Herreros et al. [26] described a new, noninvasive technique to collect midstream CCU in newborns based on bladder stimulation and lumbar paravertebral massage, with a high success rate (83.6%). Similar results were reported by Altuntas et al. (78%) [27]. However, these studies only included newborns, and the discomfort of such a technique has not been evaluated. Furthermore, the technique was performed once only, 25 minutes after feeding [26,27]. In the context of a pediatric emergency department, one would need to save time and to increase the success of urine collection by performing several attempts (e.g., before and after feeding).

There is a need for a quick, safe and effective technique for older infants, especially those admitted in emergency rooms. Although not recommended, the use of sterile bags to collect urine is still widespread, with parents quite naturally reluctant to allow the use of painful invasive techniques, particularly when the pediatrician is not certain of the diagnosis of urinary tract infection [28]. We hypothesized that the bladder stimulation technique would allow midstream urine collection without discomfort in children who are older, but still under walking age (defined in the present study as capable of taking three steps). The aim of this study was to determine the success rate of this noninvasive technique to obtain a clean-catch urine sample after two attempts, in infants under walking age. Additionally, we aimed to estimate discomfort associated with the procedure, and risk factors associated with failure of the technique.

## Materials and Methods

### Study design

This cross-sectional study was conducted between September and November 2014 in the Pediatric Emergency Department of the University Children’s Hospital of Nice (France). More than 57,000 patients are admitted to this hospital per year, of which about 3,500 infants under walking age have a urine sample taken. The study was approved by the clinical research ethics committee of the University Hospital of Nice (France [Ref 14.021]). At least one of the parents of each child gave signed consent for participation in the study after receiving verbal information from treating pediatricians.

### Technique and procedure

Bladder stimulation maneuvers were performed by only one of the four investigator physicians at a time, according to the procedure described in previous studies [26,27]. The physicians were trained to apply these maneuvers using a dummy model during the week preceding the study. The procedure involved the presence of three people. After cleaning the genital areas with warm water and soap, a nurse held the child under the armpits with legs dangling. A trained investigator physician then started bladder stimulation by gently tapping the suprapubic area at a frequency of 100 taps per minute, for 30 s. The physician then massaged the lumbar paravertebral area in the lower back for 30 s. Both maneuvers were repeated until micturition started or for a maximum of 3 minutes. We reduced the duration to a maximum of 3 minutes, in contrast to previous studies [26,27], to avoid difficulties holding older or heavier infants. A midstream urine sample was collected in a sterile container by the third person, a nurse. For illustrative purpose, a movie is available as electronic supplementary data attached to this paper (S1 File). Additional informed consent was obtained from the parents of the patient for whom identifying information is included in this article.

Success was defined as the collection of a urine sample within 3 minutes (≤180 seconds) after starting the stimulation maneuvers. If the first attempt failed, the infant was fed with water, formula or breast milk. A sterile bag was placed onto the child to avoid losing urine samples. Thirty minutes later, both maneuvers were repeated for 3 minutes. The discomfort of the technique during each attempt was evaluated using the EVENDOL score [29,30] at successively different time points of the procedure: before the maneuvers (T0), at 1 minute after the beginning of the procedure (T1), and at 1 (T2) and 5 minutes (T3) after the end of the procedure. EVENDOL, created by a French pediatric pain expert group, is a simple, validated 5-item scale (vocal or verbal expression, facial expression, movements, posture and interaction with the environment) for measuring pain in children under 8 years old in emergency departments. Items have score-bearing options ranging from “sign absent” (= 0) to “sign strong or present almost all the time” (= 3) (S1 Appendix). EVENDOL has a high level of internal consistency (Cronbach α from 0.83 to 0.92) and an excellent inter-rater reliability (weighted kappa from 0.7 to 0.9). The treatment threshold was set at 4 of 15 [29]. Thus, the event “discomfort” was evaluated as a dichotomous variable (yes/no) and was defined as an EVENDOL score above or equal to 4/15 at least once during the protocol at any time point of the procedure or during any of the attempts performed.

### Population

All infants under two years of age, not walking, and requiring urinalysis after medical advice were included. Non inclusion criteria were: presenting vital distress signs, and absence of parental consent. One of the four trained practitioners had to be present in the emergency department to inform the parents, collect their signed consent and apply the technique. Infants who voided in the sterile bag after the first attempt or infants for whom the second attempt could not be carried out (parental refusal, leaving the emergency room, or worsening of clinical status) were excluded.

### Variables

The primary outcome was the success rate for obtaining a midstream urine sample at the first or second attempt. Recorded variables included urine collection time (in seconds) defined as the elapsed time between the beginning of the stimulation procedure and the beginning of the sample collection, the final diagnosis (UTI or not), gender, age, weight, and discomfort. Diagnosis of UTI was defined, according to international recommendations [17] and previous studies, as a positive urine culture with growth of a single pathogenic organism at a concentration of ≥10^5^ colony forming units (cfu) per mL urine [28,31,32].

### Statistical analysis

Categorical variables are expressed as percentages with their 95% confidence intervals: success rate, gender (male/female), UTI (yes/no) and discomfort (yes/no). Continuous variables are expressed as mean ± standard deviation (SD), median and interquartile range (25^th^ and 75^th^ centiles): age (month), weight (kg), EVENDOL score, and time to obtain urine (s). EVENDOL scores at T0, T1, T2 and T3 for the two attempts deviated significantly from normality (tests not shown). Chi-square test or Fisher’s exact test (if n<10) were used to compare frequencies. T-test and Wilcoxon test were used to compare quantitative data according to the normality of the distribution. If a difference between two variables was statistically significant, we calculated the effect size, which is the magnitude of this difference between groups. An odds ratio (OR) was calculated to measure this effect when a Chi-square test was significant. For the same reason, a Cohen’s d was calculated if a t-test was significant. Cohen’s d was interpreted as a “small” (*d* = .2), “medium” (*d* = .5) or “large” (*d* = .8) effect size [33].

Finally, we conducted a multivariate logistic regression analysis to study associations between failure of the technique (dependent variable) and predictor variables. We considered the following as predictor variables: age (months), weight (kg), discomfort (yes/no). We adjusted analyses for gender (male/female) and UTI (yes/no) because, in addition to a higher prevalence of UTI in girls, patients with UTI may also have micturition disorders. Age and weight were kept as continuous variables in the logistic regression (hypothesis of linear modeling not rejected for both variables using nested models with the respective likelihood ratio test, 0.34 and 0.63). We then assessed the absence of co-linearity and interaction between age and weight (variance inflation factor, 4.72; p value of the interaction term, 0.09). The model was fitted by selection of variables using the Wald test. Finally, the quality of adjustment of the model was tested with Hosmer and Lemeshow’s statistic (p value, 0.57). Odds ratios are expressed with 95% confidence intervals (CI95). The significant degree for p was set at 0.05. All statistical analyses were performed using STATA version 10.0 for Macintosh^®^.

## Results

**Fig 1:**
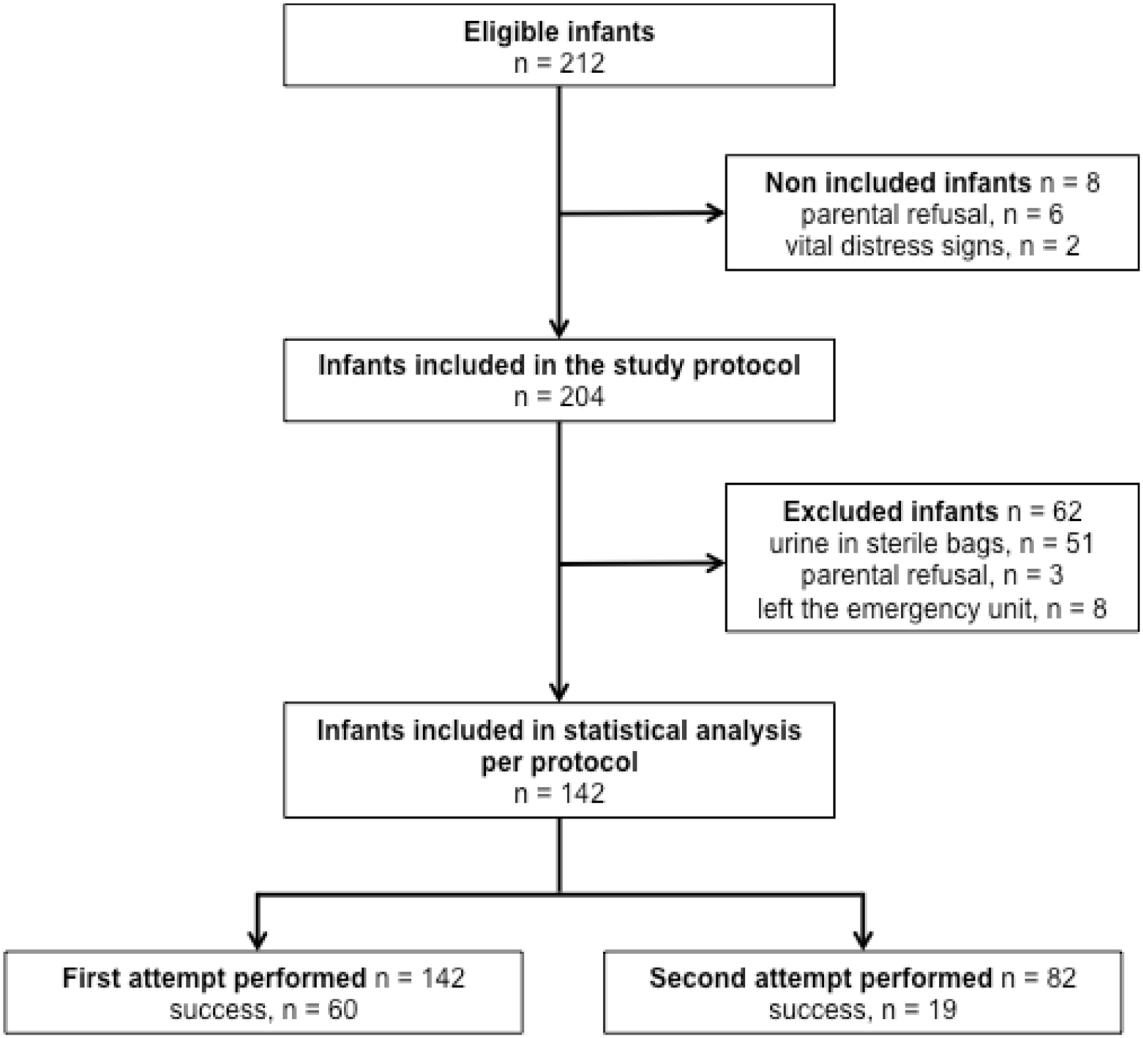
Study flow chart. Successful group, n = 79.

A total of 212 infants were eligible for the study, of which 8 were not included (Fig 1). Sixty-two infants were then excluded: 51 voided in the sterile bag after the first maneuver, 8 left the emergency department, and the parents withdrew their consent in 3 additional cases. Only one attempt was necessary for 60 infants, and two attempts for 19 infants.

The mean age and weight of the final sample (n = 142) were 4.7 months (± 4.0) and 6.2 kg (± 2.2), respectively (Table 1). The overall success rate was 55.6% (CI95 = [47.5; 63.8]), 42.3% (CI95 = [34.1; 50.4]) on the first attempt and 23.2% (CI95 = [14.0; 32.3]) on the second attempt.

**Table 1:** Clinical and procedural data.

	n	All infants (n = 142)
**Clinical data**		
Age (month)	142	4.7 (4.0)
Weight (kg)	142	6.2 (2.2)
Urinary tract infection	142	21 (14.8%)
**Procedural data**		
**Per protocol**		
Success rate	142	79 (55.6%)
^a^Time to obtain urine using bladder stimulation (s)	79	63.6 (54.9) / 52.0 (10.0;110.0)
^b^Discomfort	142	83 (58.5%)
**First attempt**		
Success rate	142	60 (42.3%)
^a^Time to obtain urine using bladder stimulation (s)	60	61.7 (55.0) / 46.0 (10.0;102.0)
Evendol score *at T0* (/15)	142	0.0 (0.0;2.0)
Evendol score *at T1* (/15)	110	6.0 (3.0;10.0)
Evendol score *at T2* (/15)	142	0.0 (0.0;3.0)
Evendol score *at T3* (/15)	142	0.0 (0.0;0.0)
^b^Discomfort	142	77 (54.2%)
**Second attempt**		
Success rate	82	19 (23.2%)
^a^Time to obtain urine using bladder stimulation (s)	19	69.7 (55.6) / 56.0 (15.0;120.0)
Evendol score *at T0* (/15)	82	0.0 (0.0;3.0)
Evendol score *at T1* (/15)	72	7.0 (3.0;10.0)
Evendol score *at T2* (/15)	82	0.0 (0.0;3.0)
Evendol score *at T3* (/15)	82	0.0 (0.0;0.0)
^b^Discomfort	82	54 (65.9%)

Values are presented as number (percentage), or mean (standard deviation, SD) and median (p25; p75) according to the variable distribution

T0: before beginning the procedure

T1: at 1 min after beginning the procedure

T2: at 1 min after the end of the procedure

T3: at 5 min after the end of the procedure

^a^ Time to obtain urine using the bladder stimulation technique is defined as elapsed time (in seconds) between the beginning of the stimulation procedure and the beginning of the sample collection

^b^ Discomfort is defined as an EVENDOL score ≥4/15 at least once during the study protocol.

The mean time to collect urine per protocol was 63.6 s (± 54.9 s), median 52.0 s (10.0; 110.0). The median times for the first and second attempt were, respectively, 46.0 s (10.0; 102.0) and 56.0 s (15.0; 120.0). As regards newborns (n = 24), the mean and median times were, respectively, 37.1 s (± 38.2 s) and 22.5 s (7.0: 58.0). The time to obtain urine between the first and the second attempt did not differ significantly (p = 0.54). The success rate for obtaining urine was not significantly different between the four investigators with the following rates: 70.0%, 51.2%, 42.9%, and 55.6% (p = 0.077). The prevalence of UTI was 14.8% (CI95 = [9.0; 20.6]) (n = 21/142). There was no statistically significant difference between boys and girls concerning age, weight, frequency of UTI, success rate, success at first or second attempt, EVENDOL score and discomfort.

**Fig 2:**
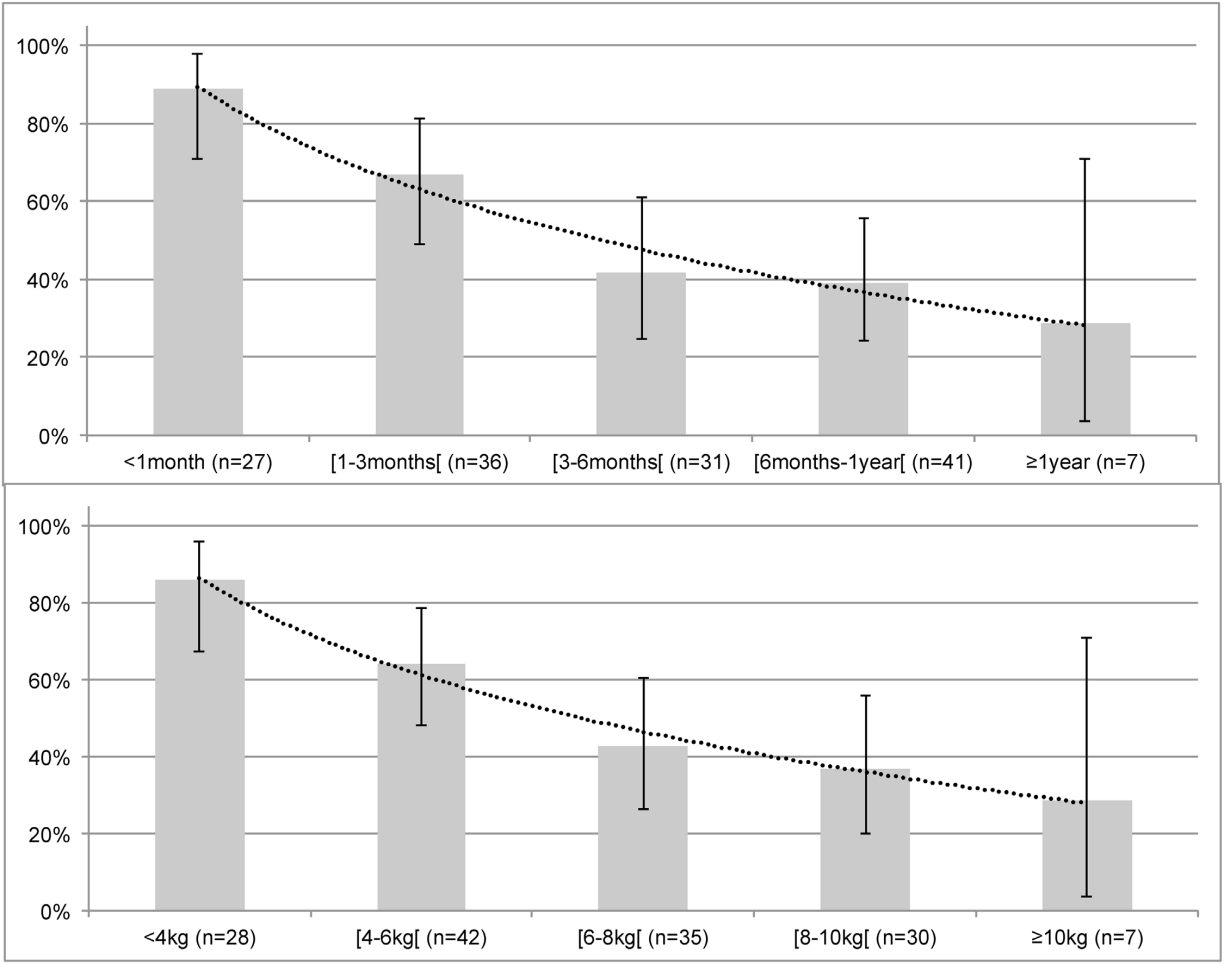
Success rate by age in months or years (top panel) and weight in kilograms (bottom panel). Success rates are presented as histograms with 95% confidence intervals (vertical line). A smooth curve (round dots) represents the success rate based on age (top panel) or on weight (bottom panel).

The success rate decreased with age (Fig 2), from 88.9% in newborns to 28.6% in infants older than 1 year (p = 0.0001), and with weight, from 85.7% for infants weighing less than 4 kg to 28.6% for infants weighing more than or equal to 10 kg (p = 0.0004).

**Fig 3:**
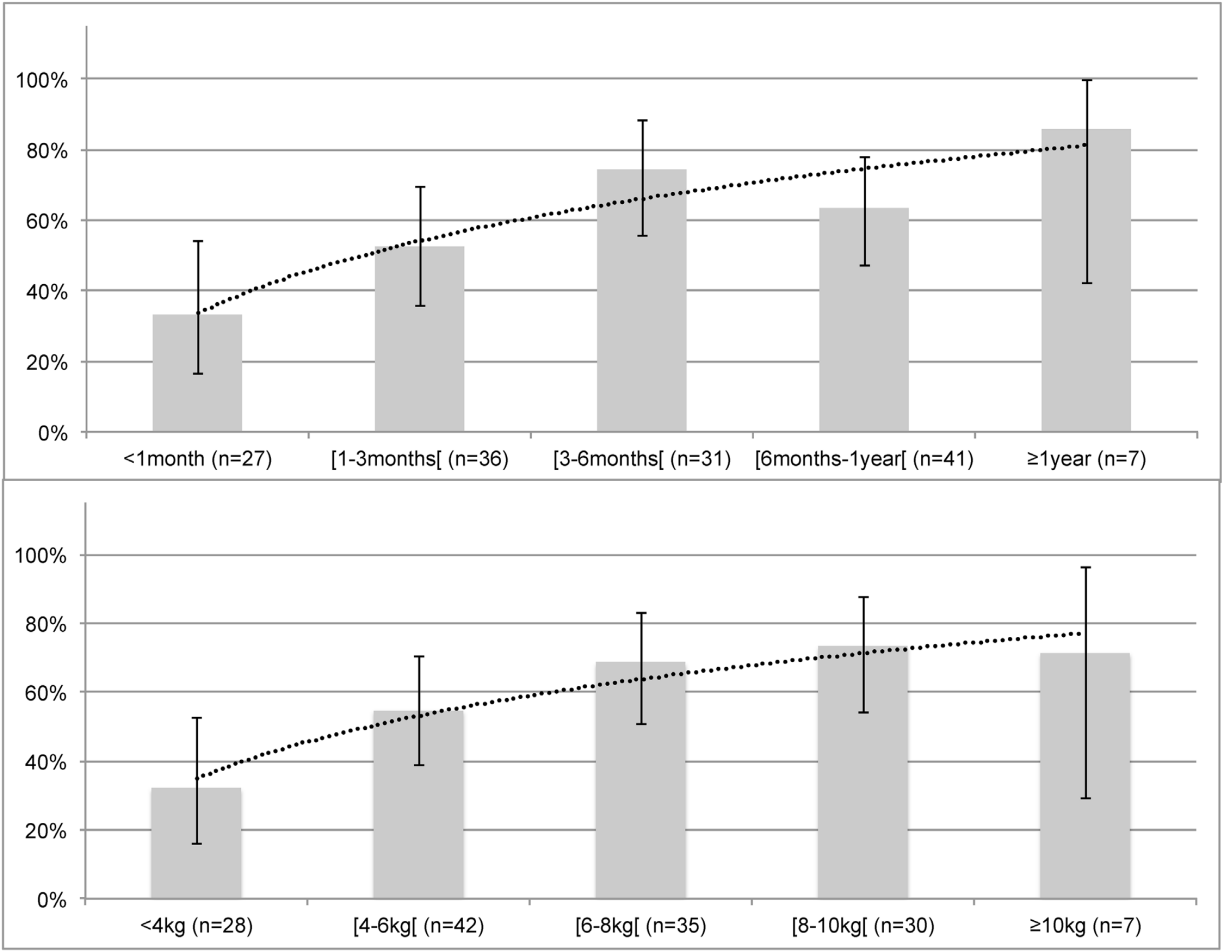
Frequency of discomfort by age in months or years (top panel) and weight in kilograms (bottom panel). Frequency of discomfort is defined as an EVENDOL score ≥4/15 at least once during the study protocol. Discomfort rates are presented as histograms with 95% confidence intervals (vertical line). The smooth curve (round dots) represents the discomfort rate based on age (top panel) or on weight (bottom panel).

During the first attempt, the median EVENDOL scores at T0, T1, T2 and T3 were, respectively, 0 (0; 2), 6 (3; 10), 0 (0; 3) and 0 (0; 0). During the second attempt, these scores were, respectively, 0 (0; 3), 7 (3; 10), 0 (0; 3) and 0 (0; 0). Among the whole study sample, 58.5% (CI95 = [50.4; 66.6]) of infants scored an EVENDOL higher than or equal to 4 at least once, and these scores at 1 minute (T2) and 5 minutes (T3) after the end of the technique remained lower than 4 for at least 75% of the infants. This prevalence significantly increased with age (p = 0.01) and weight (p = 0.012), as shown in Fig 3.

Table 2 indicates risk factors associated with failure of the procedure. In bivariate analysis, age, weight and EVENDOL score were significantly positively associated with risk of failure, with respective ORs of 1.18 (CI95 = [1.07; 1.29]), 1.44 (CI95 = [1.21; 1.71]) and 7.32 (CI95 = [3.31; 16.16]). Weight and EVENDOL score remained significantly associated with procedure failure in the multivariate analysis, with adjusted ORs of 1.47 (CI95 = [1.04; 2.06]) and 6.65 (CI95 = [2.85; 15.54]), respectively.

**Table 2:** Risk factors associated with failure of the procedure.

	Result of the procedure per protocol		Effect size	OR [CI95]	Adjusted OR [CI95]
	Success *(n = 79)*	Failure *(n = 63)*			
Age (months)†‡	3.7 (4.0)	6.0 (3.6)	Cohen’s *d* = 0.62	1.18 (1.07;1.29)	0.96 (0.81;1.15)
Weight (kg)†‡ §	5.5 (2.1)	7.1 (2.0)	Cohen’s *d* = 0.79	1.44 (1.21;1.71)	1.47 (1.04;2.06)
Gender					
Girl	44 (55.7%)	30 (47.6%)	OR = 0.73 (0.35;1.48)	1	1
Boy	35 (44.3%)	33 (52.4%)		1.38 (0.71;2.69)	1.71 (0.77;3.79)
Urinary tract infection					
No	65 (82.3%)	56 (88.9%)	OR = 1.72 (0.60;5.40)	1	1
Yes	14 (17.7%)	7 (11.1%)		0.58 (0.22;1.54)	0.79 (0.26;2.40)
^a^Discomfort†‡§					
No	48 (60.8%)	11 (17.5%)	OR = 0.14 (0.06;0.32)	1	1
Yes	31 (39.2%)	52 (82.5%)		7.32 (3.31;16.16)	6.65 (2.85;15.54)

Values are presented as number (row percentage), or mean (SD) and median (p25; p75)

Risks are presented as odds ratios and 95% confidence intervals

^†^ p<0.05 (by result of the procedure)

^‡^ p<0.05 (univariate analysis)

^§^ p<0.05 (multivariate analysis)

^a^Discomfort was defined as an Evendol score ≥4/15 at least once during the study protocol.

## Discussion

The bladder stimulation technique has been described recently in neonates [26,27]. Our study is the first using this technique in infants in a large pediatric emergency unit. The maneuvers of bladder stimulation were carried out by only one of the four investigator physicians trained, avoiding inter-individual variability in performance of these maneuvers and leading to a high strength of the present study. However, this technique may not succeed for all infants. The results suggest that heavy weight and discomfort were both significantly associated with failure of the technique.

In the whole population, the success rate was 55.6% and the median time necessary to sample urine was 52 s. However, the success rate was higher in younger infants with an 88.9% success rate during the first month of life (n = 24), with mean and median urine collection times of 37.1 s (± 38.2) and 22.5 s (7.0; 58.0), respectively. These results are similar to those of the studies of Herrera et al. and Altuntas et al. in newborns [26,27]. We found, however, that the success rate remained high, at 64.9% (CI95 = [55.2; 74.5]) among infants less than 6 months of age, suggesting that a midstream urine sample may be collected using the bladder stimulation method in infants older than 1 month.

Micturition is spontaneous during infancy. It involves several muscles of different categories (striated and smooth) and requires integrity of the central and autonomic nervous systems to obtain the innate voiding reflex. Two sacral nerves are important in bladder function: the pudendal nerve and the pelvic splanchnic nerve. Bladder emptying by a sacral reflex is present as early as the twentieth week of gestational age [34]. At birth, 20–24 micturition episodes per day are induced by proprioceptive stimulus of bladder straining and also by the pudendal reflex. Until 2–3 years of age, the spinal reflex is progressively inhibited by influxes from higher spinal centers. However, before 2 years of age, the guarding reflex to avoid micturition is not controlled and the pudendal nerve is unable to avoid micturition in the case of increased abdominal pressure [35] or bladder stimulation. The activation of pudendal afferents can evoke reflex bladder contraction or relaxation, depending on the frequency of stimulation and the filling of the bladder, but the pathway and center for this pudendal reflex are unknown. It is responsible for micturition in infants after stimulation (cold, heat, drying) of the perineal skin or local inflammation (foreskin adhesion, vaginitis, perineal dermatitis induced by skin maceration, etc.).

We expected that our study protocol would not be able to be respected in some patients. Indeed, 51 infants were excluded from the analysis because they urinated in sterile bags before the second attempt (Fig 1). If all patients from who urine samples were collected (n = 193) are included in the analysis, an alternate calculation of the success rate is 40.9% (CI95 = [33.9; 48.2]). However, this low alternate success rate may be explained by the fact that we decided not to reproduce identically the protocol described in Herrera’s study. First, we stopped the technique after 3 minutes and not 5 minutes. Secondly, if the infant urinated in a sterile bag between attempts, it was not conceivable to collect urine again using an experimental and potentially painful technique. Placing and removing the bag may be as distressing as the bladder stimulation technique. In addition if the urinalysis from a bag-collected sample is positive, a technique with a lower risk of contamination should be used [17].

The bladder stimulation technique provides midstream clean-catch urine for the diagnosis of UTI, as recommended by the American Academy of Pediatrics in infants after the age of toilet training and in adults. However, we did not evaluate the performance of this technique compared to a gold standard. Following our usual practice in this diagnosis, only 52 urine cultures were obtained using this technique. Of this subgroup, 20 urine cultures (40%) were either a misdiagnosis or an impossible diagnosis. According to our study protocol, we did not focus only on the UTI diagnosis and we included all patients for whom a urinalysis was needed. Thus, our misdiagnosis rate was probably overestimated. Altuntas et al estimated a contamination rate of 24% [27] using this technique in neonates but they did not evaluate the performance using a gold standard (suprapubic aspiration or bladder catheterization).

The discomfort of the technique was documented at different times of the procedure with the hypothesis that discomfort may remain until 5 minutes after the end of the procedure. An EVENDOL score higher than or equal to 4 was obtained at least once for 58.5% of infants. Our results indicated that discomfort occurred mainly during the procedure, but did not persist after the end of the technique. In addition, we found that discomfort is a risk factor for failure of the technique (adjusted OR = 6.65 (CI95 = [2.85; 15.54])). The EVENDOL score increased with age and weight (Fig 3) and heavy weight was positively associated with a risk of failure (adjusted OR = 1.47 (CI95 = [1.04; 2.06])). This result could be explained by the difficulty of correctly holding a heavy infant, thus the infant may be uncomfortable, not allowing us to perform the technique in good conditions. This side effect may be improved by using a system of holding infants without discomfort. For instance, distraction techniques or music that are usually used during painful procedures in pediatric patients could be used during the bladder stimulation technique to reduce the child’s discomfort. Nevertheless, bladder stimulation provides midstream CCU, a significant advantage compared to the discomfort it causes.

Finally, the procedure using bladder stimulation may be a faster method to obtain urine for diagnostic purposes, but further randomized study is needed to control this result.

We conclude that the bladder stimulation technique is easy to perform, noninvasive, time sparing and gives good results in younger infants. It could be proposed as an alternative to other urine collection techniques.

Our promising results have several limitations. Although conducted in a large pediatric emergency unit, this was a single center study and thus the results need to be confirmed by others. Trained staff are required to perform such a technique and only four medical investigators from our staff are presently trained in this technique, thus our experience is limited to the presence of these personnel. We need to train other investigators (on low-fidelity mannequins) to allow larger studies. Moreover, this technique required the presence two people (medical staff members) in addition to the investigator. Our success with this procedure was probably dependent on the organization of our medical team in order to have enough available people trained, which is not usual in pediatric emergency units.

Furthermore, we lack a randomized control group to compare the efficiency of this technique with others. Nevertheless, our results are similar to those of previous studies [26,27] and suggest that the technique can be used to collect midstream urine quickly.

This technique has been implemented successfully in our center. However, further large-scale studies are necessary to evaluate the efficiency, side effects (discomfort) and gain of time associated with this technique compared with other urine collection methods.

## Conclusion

A new technique of bladder stimulation and paravertebral massage, described as a method to collect midstream clean-catch urine in newborns, is highly successful in infants younger than 6 months. However, the results of the EVENDOL scores showed that the procedure causes mild to moderate short-duration distress during the procedure. There is a need to reduce the discomfort associated with this procedure in order to facilitate urine collection. This technique may be an alternative procedure to obtain a urine sample, avoiding the utilization of other time-consuming, inaccurate (e.g., bag) or invasive urine collection methods. However, further studies are needed to validate this procedure before it is implemented in pediatric emergency units.

## Supporting Information

S1 Appendix EVENDOL score. (PDF)

S1 Checklist (DOC)

S1 File Bladder stimulation and paravertebral massage. (M4V)
