# Supplementary material for: Evaluation of the Bladder Stimulation Technique to Collect Midstream Urine in Infants in a Pediatric Emergency Department
Source: PLoS One. 2016 Mar 31;11(3):e0152598. doi: 10.1371/journal.pone.0152598 (PMC4816310; doi:10.1371/journal.pone.0152598)
Supplement: S1 Appendix — (PDF) [file pone.0152598.s001.pdf]

Evaluation Enfant Douleur

EVENDOL

Echelle validée
de la naissance à 7 ans.

Score de 0 à 15,
seuil de traitement 4/15.

Notez tout ce que vous observez... même si vous pensez que les signes ne sont pas dus à la douleur, mais à la peur, à l'inconfort, à la fatigue ou à la gravité de la maladie.

Nom	Signe **absent**	Signe **faible ou passager**	Signe **moyen ou environ la moitié du temps**	Signe **fort ou quasi permanent**	**Evaluation à l'arrivée**		Antalgique ▼ **Evaluations suivantes Evaluations après antalgique**[3]			
					au repos[1] au calme (R)	à l'examen[2] ou la mobilisation (M)	R / M	R / M	R / M	R / M
Expression vocale ou verbale										
**pleure *et/ou* crie *et/ou* gémit *et/ou* dit qu'il a mal**	0	1	2	3						
Mimique										
**a le front plissé *et/ou* les sourcils froncés *et/ou* la bouche crispée**	0	1	2	3						
Mouvements										
**s'agite *et/ou* se raidit *et/ou* se crispe**	0	1	2	3						
Positions										
**a une attitude inhabituelle *et/ou* antalgique *et/ou* se protège *et/ou* reste immobile**	0	1	2	3						
Relation avec l'environnement										
**peut être consolé *et/ou* s'intéresse aux jeux *et/ou* communique avec l'entourage**	normale 0	diminuée 1	très diminuée 2	absente 3						
Remarques				**Score total /15**						
				Date et heure						
				Initiales évaluateur						

[1] Au repos au calme (R) : observer l'enfant avant tout soin ou examen, dans les meilleures conditions possibles de confort et de confiance, par exemple à distance, avec ses parents, quand il joue...
[2] A l'examen ou la mobilisation (M) : il s'agit de l'examen clinique ou de la mobilisation ou palpation de la zone douloureuse par l'infirmière ou le médecin.
[3] Réévaluer régulièrement en particulier après antalgique, au moment du pic d'action : après 30 à 45 minutes si oral ou rectal, 5 à 10 minutes si IV. Préciser la situation, au repos (R) ou à la mobilisation (M).

Echelle validée pour mesurer la douleur (aiguë ou prolongée avec atonie), de 0 à 7 ans, en pédiatrie, aux urgences, au SAMU, en salle de réveil, en post-opératoire - Référence bibliographique : Archives de Pédiatrie 2006, *13*, 922, P129-130. Archives de Pédiatrie 2012, *19*, 922, P42-44. *Journées Paris Pédiatrie 2009 : 265-276. Pain 2012, 153 : 1573-1582.* Contact : elisabeth.fournier-charriere@bct.aphp.fr - © 2011 - Groupe EVENDOL

Zid et Zen communication - 01 46 49 96 79 - 08/12

Evaluation Enfant Douleur

EVENDOL

Echelle validée de la naissance à 7 ans.

Score de 0 à 15, seuil de traitement 4/15.

Notez tout ce que vous observez... même si vous pensez que les signes ne sont pas dus à la douleur, mais à la peur, à l'inconfort, à la fatigue ou à la gravité de la maladie.

Nom	Signe absent	Signe **faible** ou passager	Signe **moyen ou environ la moitié du temps**	Signe **fort ou quasi permanent**	**Evaluations suivantes - Evaluations après antalgique**[3]							
					R[1] / M[2]	R / M	R / M	R / M	R / M	R / M	R / M	R / M
Expression vocale ou verbale												
**pleure *et/ou* crie *et/ou* gémit *et/ou* dit qu'il a mal**	0	1	2	3								
Mimique												
**a le front plissé *et/ou* les sourcils froncés *et/ou* la bouche crispée**	0	1	2	3								
Mouvements												
**s'agite *et/ou* se raidit *et/ou* se crispe**	0	1	2	3								
Positions												
**a une attitude inhabituelle *et/ou* antalgique *et/ou* se protège *et/ou* reste immobile**	0	1	2	3								
Relation avec l'environnement												
**peut être consolé *et/ou* s'intéresse aux jeux *et/ou* communique avec l'entourage**	normale 0	diminuée 1	très diminuée 2	absente 3								
Remarques				**Score total /15**								
				Date et heure								
				Initiales évaluateur								

[1] Au repos au calme (R) : observer l'enfant avant tout soin ou examen, dans les meilleures conditions possibles de confort et de confiance, par exemple à distance, avec ses parents, quand il joue...
[2] A l'examen ou la mobilisation (M) : il s'agit de l'examen clinique ou de la mobilisation ou palpation de la zone douloureuse par l'infirmière ou le médecin.
[3] Réévaluer régulièrement en particulier après antalgique, au moment du pic d'action : après 30 à 45 minutes si oral ou rectal, 5 à 10 minutes si IV. Préciser la situation, au repos (R) ou à la mobilisation (M).

Echelle validée pour mesurer la douleur (aiguë ou prolongée avec atonie), de 0 à 7 ans, en pédiatrie, aux urgences, au SAMU, en salle de réveil, en post-opératoire - Référence bibliographique : Archives de Pédiatrie 2006, *13*, 922, P129-130. Archives de Pédiatrie 2012, *19*, 922, P42-44. *Journées Paris Pédiatrie 2009 : 265-276. Pain 2012, 153 : 1573-1582.* Contact : elisabeth.fournier-charriere@bct.aphp.fr - © 2011 - Groupe EVENDOL

Zid et Zen communication - 01 46 49 96 79 - 08/12
