# Supplementary material for: Evaluation of the Bladder Stimulation Technique to Collect Midstream Urine in Infants in a Pediatric Emergency Department
Source: PLoS One. 2016 Mar 31;11(3):e0152598. doi: 10.1371/journal.pone.0152598 (PMC4816310; doi:10.1371/journal.pone.0152598)
Supplement: S1 Checklist — (DOC) [file pone.0152598.s002.doc]

STROBE Statement—checklist of items that should be included in reports of observational studies

|  | Item No. | Recommendation | Page  No. | Relevant text from manuscript |
| --- | --- | --- | --- | --- |
| **Title and abstract** | 1 | (*a*) Indicate the study’s design with a commonly used term in the title or the abstract | 2 | […]a cross sectional study […] |
|  |  | (*b*) Provide in the abstract an informative and balanced summary of what was done and what was found | 2 |  |
| Introduction | | | |  |
| Background/rationale | 2 | Explain the scientific background and rationale for the investigation being reported | 4 | […] midstream clean catch urine (CCU) is an accepted method to diagnose UTI.[…]. The natural voiding pattern […]. Herreros et Al described a new, noninvasive technique […] with a high success rate. |
| Objectives | 3 | State specific objectives, including any prespecified hypotheses | 5 | We hypothesized that […]  The aim of this study was […] |
| Methods | | | |  |
| Study design | 4 | Present key elements of study design early in the paper | 6 | This cross-sectional study […] |
| Setting | 5 | Describe the setting, locations, and relevant dates, including periods of recruitment, exposure, follow-up, and data collection | 6 | […]between September and November 2014 […] Pediatric Emergency Department of […] |
| Participants | 6 | (*a*) *Cohort study*—Give the eligibility criteria, and the sources and methods of selection of participants. Describe methods of follow-up  *Case-control study*—Give the eligibility criteria, and the sources and methods of case ascertainment and control selection. Give the rationale for the choice of cases and controls  *Cross-sectional study*—Give the eligibility criteria, and the sources and methods of selection of participants | 7 | Population |
|  |  | (*b*) *Cohort study*—For matched studies, give matching criteria and number of exposed and unexposed  *Case-control study*—For matched studies, give matching criteria and the number of controls per case |  | Not applicable |
| Variables | 7 | Clearly define all outcomes, exposures, predictors, potential confounders, and effect modifiers. Give diagnostic criteria, if applicable | 8 | Variables section |
| Data sources/ measurement | 8* | For each variable of interest, give sources of data and details of methods of assessment (measurement). Describe comparability of assessment methods if there is more than one group | 8 | Variables section |
| Bias | 9 | Describe any efforts to address potential sources of bias | 9 | […] gender and UTI […] |
| Study size | 10 | Explain how the study size was arrived at | 9 | A total of 212 infants […] |

Continued on next page

| Quantitative variables | 11 | Explain how quantitative variables were handled in the analyses. If applicable, describe which groupings were chosen and why | 9 | Age and weight were kept as continuous variables […] |
| --- | --- | --- | --- | --- |
| Statistical methods | 12 | (*a*) Describe all statistical methods, including those used to control for confounding | 8,9 | EVENDOL scores at […] deviated significantly from normality (tests not shown)  Chi-square test or […]  T-test and […]  […] we calculated the effect sizes  […] multivariate logistic regression […]  We then assessed the absence of collinearity and interaction […]  […] Wald test  […] Hosmer and Lemeshow’s statistic […]  Odds ratios […] (CI95) |
|  |  | (*b*) Describe any methods used to examine subgroups and interactions | 9 | […]We then assessed the absence of collinearity and interaction […] nested models […] |
|  |  | (*c*) Explain how missing data were addressed |  | There were no missing data. There were 4 investigators who performed the technique. There were all Medical Doctor and only a few data were collected. That explained that only 212 infants were eligible compared to the size of the unit |
|  |  | (*d*) *Cohort study*—If applicable, explain how loss to follow-up was addressed  *Case-control study*—If applicable, explain how matching of cases and controls was addressed  *Cross-sectional study*—If applicable, describe analytical methods taking account of sampling strategy |  | Not applicable |
|  |  | (*e*) Describe any sensitivity analyses |  | Not applicable |
| Results | | | | |
| Participants | 13* | (a) Report numbers of individuals at each stage of study—eg numbers potentially eligible, examined for eligibility, confirmed eligible, included in the study, completing follow-up, and analysed | 9 | A total of 212 infants […] |
|  |  | (b) Give reasons for non-participation at each stage | 9 | 51 voided […], 8 left [..], withdrew their consent in 3 additional cases. |
|  |  | (c) Consider use of a flow diagram | 9 | Fig 1 |
| Descriptive data | 14* | (a) Give characteristics of study participants (eg demographic, clinical, social) and information on exposures and potential confounders | 10 | Table 1 |
|  |  | (b) Indicate number of participants with missing data for each variable of interest |  | There were no missing data. |
|  |  | (c) *Cohort study*—Summarise follow-up time (eg, average and total amount) |  | Not applicable |
| Outcome data | 15* | *Cohort study*—Report numbers of outcome events or summary measures over time |  | Not applicable |
|  |  | *Case-control study—*Report numbers in each exposure category, or summary measures of exposure |  | Not applicable |
|  |  | *Cross-sectional study—*Report numbers of outcome events or summary measures | 10  11  11  12  13 | The overall success rate was 55.6% […].  The mean time […] 63.6 s […]  The prevalence of UTI xas 14.8% […]  Fig 2  During the first attempt, the median EVENDOL scores at […] were, respectively, 0 (0; 2), 6 (3; 10), 0 (0; 3) and 0 (0; 0). During the second attempt […] respectively, 0 (0; 3), 7 (3; 10), 0 (0; 3) and 0 (0; 0).  […] as shown in Fig 3.  In bivariate analysis, age, weight and EVENDOL score […] respective OR of 1.18 (CI95 = [1.07; 1.29]), 1.44 (CI95 = [1.21; 1.71]) and 7.32 (CI95 = [3.31;  16.16]). Weight and EVENDOL […] in multivariate analysis, adjusted ORs of 1.47 (CI95 = [1.04; 2.06]) and 6.65 (CI95 = [2.85; 15.54]) respectively  Table 2. Risk factors […] |
| Main results | 16 | (*a*) Give unadjusted estimates and, if applicable, confounder-adjusted estimates and their precision (eg, 95% confidence interval). Make clear which confounders were adjusted for and why they were included | 9  12  13 | […] multivariate logistic regression analysis to study associations between failure of the technique (dependent variable) and predictor variables. We considered the following as predictor variables: age (months), weight (kg), discomfort (yes/no). We adjusted the analyses for gender (male/female) and UTI (yes/no). […]  In bivariate analysis, age, weight and EVENDOL score […] respective OR of 1.18 (CI95 = [1.07; 1.29]), 1.44 (CI95 = [1.21; 1.71]) and 7.32 (CI95 = [3.31;  16.16]). Weight and EVENDOL […]in multivariate analysis, adjusted OR of 1.47 (CI95 = [1.04; 2.06]) and 6.65 (CI95 = [2.85; 15.54]) respectively  Table 2. Risk factors […] |
|  |  | (*b*) Report category boundaries when continuous variables were categorized | 7 | EVENDOL score […] Thus, the event “discomfort” was evaluated as a dichotomous variable (yes/no) and was defined as an EVENDOL score above or equal to 4/15 at least once during the protocol […] |
|  |  | (*c*) If relevant, consider translating estimates of relative risk into absolute risk for a meaningful time period |  | Not applicable |

Continued on next page

| Other analyses | 17 | Report other analyses done—eg analyses of subgroups and interactions, and sensitivity analyses | 10  11  11 | The overall success rate was 55.6% […].  The mean time […] 63.6 s […]  The prevalence of UTI xas 14.8% […]  Fig 2  During the first attempt, the median EVENDOL scores at […] were, respectively, 0 (0; 2), 6 (3; 10), 0 (0; 3) and 0 (0; 0). During the second attempt […]respectively, 0 (0; 3), 7 (3; 10), 0 (0; 3) and 0 (0; 0).  […] as shown in Fig 3. |
| --- | --- | --- | --- | --- |
| Discussion | | | | |
| Key results | 18 | Summarise key results with reference to study objectives | 14  16 | In the whole population, the success rate was 55.6% and the median time necessary to sample urine was 52 s  we found that discomfort is a risk factor for failure of the technique (adjusted OR = 6.65 (CI95 = [2.85; 15.54])). The EVENDOL score increased with age and weight (Fig 3) and heavy weight was positively associated with a risk of failure (adjusted OR = 1.47 (CI95 = [1.04; 2.06])). |
| Limitations | 19 | Discuss limitations of the study, taking into account sources of potential bias or imprecision. Discuss both direction and magnitude of any potential bias | 17 | […] Although conducted in a large pediatric emergency unit, this was a single center study […] we lack a randomized control group to compare the efficiency of this technique with others |
| Interpretation | 20 | Give a cautious overall interpretation of results considering objectives, limitations, multiplicity of analyses, results from similar studies, and other relevant evidence | 15  16 | We expected that our study protocol would not be able to be respected […] an alternate calculation of the success rate is 40.9% (CI95 = [33.9; 48.2]).  […] our misdiagnosis rate is probably overestimated […] |
| Generalisability | 21 | Discuss the generalisability (external validity) of the study results | 17 | […]single center study […]Trained staff are required […]dependent on the organization of our medical team […] we lack a randomized control group to compare the efficiency of this technique with others |
| Other information | |  | | |
| Funding | 22 | Give the source of funding and the role of the funders for the present study and, if applicable, for the original study on which the present article is based |  | There was no source of funding |

*Give information separately for cases and controls in case-control studies and, if applicable, for exposed and unexposed groups in cohort and cross-sectional studies.

**Note:** An Explanation and Elaboration article discusses each checklist item and gives methodological background and published examples of transparent reporting. The STROBE checklist is best used in conjunction with this article (freely available on the Web sites of PLoS Medicine at http://www.plosmedicine.org/, Annals of Internal Medicine at http://www.annals.org/, and Epidemiology at http://www.epidem.com/). Information on the STROBE Initiative is available at www.strobe-statement.org.
